# Correlated noise in Brownian motion allows for super resolution

**DOI:** 10.1038/s41598-020-76745-4

**Published:** 2020-11-12

**Authors:** Santiago Oviedo-Casado, Amit Rotem, Ramil Nigmatullin, Javier Prior, Alex Retzker

**Affiliations:** 1 Racah Institute of Physics, The Hebrew University of Jerusalem, 91904 Jerusalem, Givat Ram Israel; 2 Complex Systems Research Group and Centre for Complex Systems, Faculty of Engineering and IT, The University of Sydney, Sydney, NSW 2006 Australia; 3 Área de Física Aplicada, Universidad Politécnica de Cartagena, 30202 Cartagena, Spain; 4 Instituto Carlos I de Física Teórica y Computacional, Universidad de Granada, 18071 Granada, Spain

**Keywords:** Quantum physics, Quantum metrology

## Abstract

Diffusion broadening of spectral lines is the main limitation to frequency resolution in non-polarized liquid state nano-NMR. This problem arises from the limited amount of information that can be extracted from the signal before losing coherence. For liquid state NMR as with most generic sensing experiments, the signal is thought to decay exponentially, severely limiting resolution. However, there is theoretical evidence that predicts a power law decay of the signal’s correlations due to diffusion noise in the non-polarized nano-NMR scenario. In this work we show that in the NV based nano-NMR setup such diffusion noise results in high spectral resolution.

## Introduction

Spectral analysis is of utmost importance in a wide variety of fields from material science, to biology and medicine. Among the most widespread techniques to obtain structural information in the form of a spectrum is Nuclear Magnetic Resonance (NMR), which is nonetheless hindered by low sensitivity. One promising approach to improve the capacities of NMR is to reduce the sample to the nano-scale. This technique, however, is still limited by the finite resolution of spectral features. A possible solution is to use polarized samples as in conventional NMR^1,2^, but this approach requires either large samples or a substantial increase in experimental complexity. In this work we challenge the claim that working with nano-sized samples limits resolution, and provide analytical and numerical evidence supporting the viability of the non-polarized setup as an alternative route to nano-NMR.

NV centers have been used extensively in the past as quantum sensors for the implementation of the nano-NMR scheme^1,3–10^. In particular, the use of quantum heterodyne (Qdyne) measurement techniques (know as well as synchronized measurements), together with a suitable data-analysis algorithm has demonstrated that resolving two close frequencies requires no more than accumulating a sufficient number of measurements^11,12^. These techniques, however, are computationally heavy since they need to solve a global maximization problem in a large dimensional space that grows linearly with measurement time.

**Figure 1:**
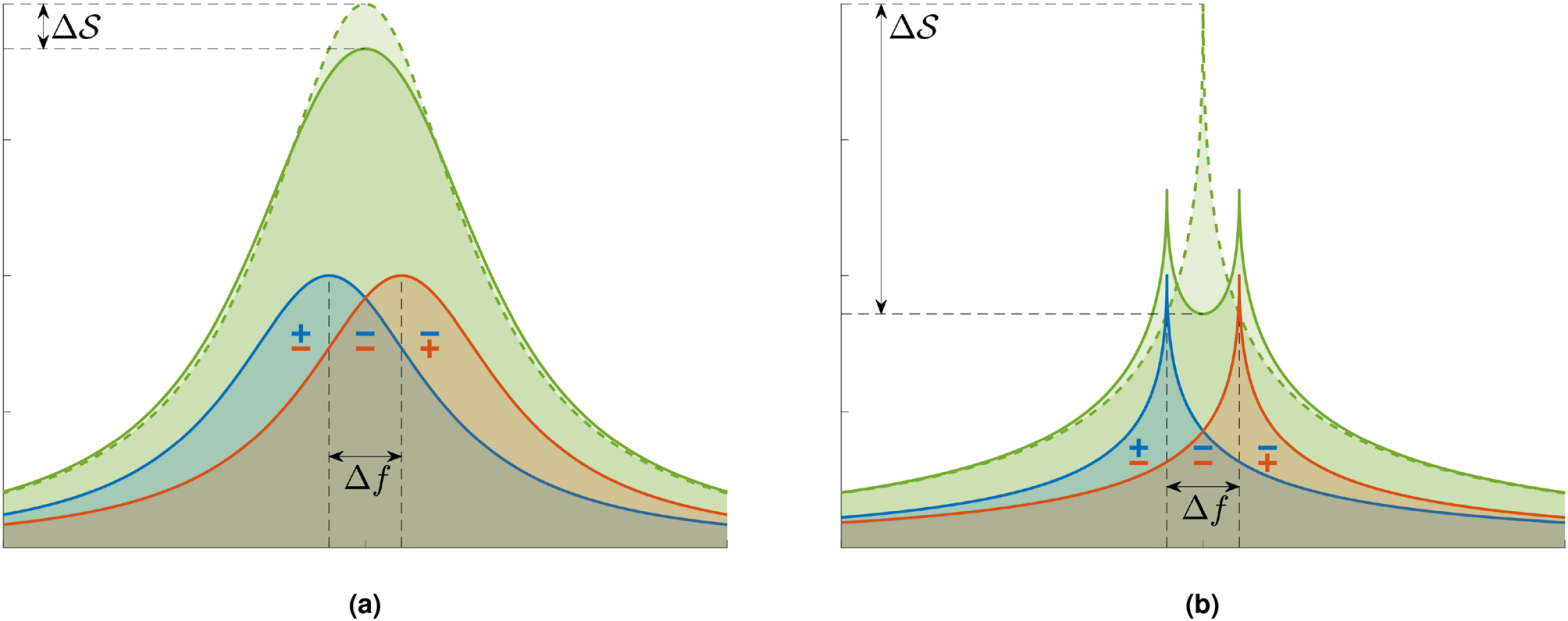
Problem illustration. When the line-shapes of two underlying frequencies (blue and orange) overlap, the measured line-shape (solid green) can be very similar to line-shape of a single, strong frequency (dashed green). The difference between the two line-shapes is most notable at the peak/center of the spectrum, where the changes brought about by the two underlying line-shapes coincide (blue and orange “minus” signs, indicating that $\Delta \mathscr {S}$ is negative for a finite $\Delta f$), whereas at the edge of the spectrum the changes are opposite (blue “plus” and orange “minus” signs on the left, and vice versa on the right). **(a)** For a smooth function; e.g., Gaussian or Lorentzian, $\Delta \mathscr {S} / \Delta f$ is linear in $\Delta f$ and thus small. In contrast, for a sharp-peak function as in **(b)** $\Delta \mathscr {S} / \Delta f \sim \Delta f^{-1/2}$, as can be shown from the diffusion dominated correlation function (Eq. 13), and resolution is not limited. See Supplementary Information for more details.

Measuring a spectrum that contains two (or more) similar frequencies that are closer than the characteristic width of their line-shape results in a resolution problem (Fig. 1). The intuition behind the limited resolution can be understood in terms of the Rayleigh criterion from optics, where two images are resolvable only up to the wavelength used to image them. Here, the width of the line-shape plays the role of the wavelength. This resolution problem for two close frequencies can best be understood by looking at the change in the spectrum ($\mathscr {S}$) as a function of the frequency difference. For a smooth function; e.g., a Lorentzian, a finite frequency difference has a very small effect on the spectrum (Fig. 1a), whereas for a sharp-peak function the change is more pronounced (Fig. 1b). This suggests that for a sharp-peaked spectrum, spectral-resolution could be improved.

Spectral resolution in NV based liquid-state nano-NMR is limited mainly by the diffusion of nuclei in the sample^13–16^. When measuring a noisy signal oscillating at frequency $\delta$, the amount of information that can be extracted from the auto-correlation of the signal; e.g., $\cos (\delta t)C(t)$, is limited by the noise coherence time. For diffusion noise in liquid state nano-NMR, $C(t)$ is generally considered to be an exponentially decaying function leading to Lorentzian spectral line-shapes, impeding high spectral resolution. In this manuscript, we challenge this framework by building on the work of Cohen et al.^17^, which reported that a significant deviation from the Lorentzian line-shape paradigm occurs when measuring a magnetic field of a non-polarized nano-sized liquid sample with a shallow NV. We show that diffusion does not limit resolution and that the analysis is computationally amenable and can be done with simple algorithms such as Fourier spectrum analysis.

The effect in^17^ can be understood as follows. The effective sensitivity of an NV located at depth $d$ beneath a sample extends to a semi-sphere of radius $d$ above the surface that contains $N\propto d^3$ non-polarized nuclei. The rms of the magnetic field sensed by the NV is thus $B_{\mathrm{rms}}\propto \sqrt{N}/d^3$, where the $d^3$ is due to the dipole-dipole interaction between NV and nuclei. The peak of the power spectrum is thus $S(\delta = 0) \propto B_{\mathrm{rms}}^2 T_\phi \propto 1/d$, with $T_\phi \propto d^2$ the characteristic time that it takes the nuclei to diffuse out of the semi-sphere (i.e., the inverse of the signal bandwidth). When, for example, applying dynamical decoupling (DD) sequence with detuning $\delta$ from the nuclei Larmor frequency, a new length scale is introduced, i.e., $\ell =\sqrt{D/\delta }$; this length scale can be understood as a cut-off for the interaction between NV and distant nuclei; fields coming from these nuclei are slow changing and thus attributed to low frequency. Using the same reasoning as before, the power spectrum around the peak is $S(\delta ) \propto 1/d-\alpha /\ell = 1/d - \alpha \sqrt{\delta / D}$, where $\alpha$ is a positive number. Therefore the power spectrum in NV based nano-NMR of liquid samples is a sharp-peaked function. A similar effect has also been observed in diffusing atom systems^18^. Conversely, in the time domain, where the resolution problem is manifested by our ability to see a beat-note, the measurement protocol with a shallow NV produces a correlation function with polynomial rather than exponential decay, such that the beating between close frequencies can be observed, allowing higher resolution.

## Results

### FI analysis

We now analyze the effect of long-lived correlations on frequency estimation and resolution. The resolution problem is characterized by an estimation error for the frequencies that diverges when the frequency difference is much smaller than the characteristic noise frequency, $T_\phi ^{-1}$, as demonstrated by a vanishing amount of information extracted from the signal^19^. For a noise that is a stationary Gaussian process, with a covariance function of the form $\text {Cov}( t ) \propto C( t ) \sum _{j=1}^N \cos ( \delta _j t )$, the resolution problem occurs for $| \delta _i -\delta _j | T_\phi < 1$. We restrict the derivation to the estimation of a small single frequency, which is a good model for the resolution problem since the average frequency is generally easier to estimate. We analyze the three possible measurement scenarios, i.e. correlation spectroscopy^4,20^, Qdyne/synchronized measurement protocol^1,8,9^, and power spectrum probing^21^. For the full details of this derivation and schematics of each protocol we refer the reader to the Supplementary Information.

#### Correlation spectroscopy

The fluorescence response of the NV can be modeled by a Poisson distribution with a rate parameter that depends on the NV state ($m=0,1$). In the correlation spectroscopy scenario, the average number of photons detected is given by^4,20^

1 $$\begin{aligned} p = \eta + \frac{c}{2} \langle \sin (\phi _s ) \sin (\phi _{s+t} ) \rangle , \end{aligned}$$

where $\eta ,c$ are the average detection rate and contrast, and $\phi _s (\phi _{s+t})$ is the phase accumulated by the NV during the first (second) interrogation time ($\tau$). These phases are calculated by integrating over the magnetic field. We model the magnetic field as stationary Gaussian processes oscillating at frequency $\delta$, with a characteristic correlation time $T_\phi$ and a mean field strength of $B_\text {rms}$. Averaging over realizations of the magnetic field yields

2 $$\begin{aligned} p = \eta + \frac{c}{2} e^{-\phi _{\text {rms}}^2} \sinh (\phi _\text {rms}^2 \cos ( \delta t ) C (t/T_\phi ) ), \end{aligned}$$

where $C( \cdot )$ is the correlation function (envelope) of the phases. The rms of the accumulated phase and its correlation function can be approximated by $\phi _\text {rms} \approx \gamma B_\text {rms}\tau$ and $C(t/T_\phi )\cos ( \delta t ) \approx \text {corr}(B_s,B_{s+t})$ for a short interrogation time $\tau \ll T_\phi$, where $\gamma$ is the gyromagnetic ratio of the NV. For a weak signal (i.e., $\phi _\text {rms}^2 \ll 1$) Eq. (2) can be approximated by

3 $$\begin{aligned} p \approx \eta + \frac{c}{2} \phi _{\text {rms}}^2 \cos ( \delta t ) C( t/T_\phi ). \end{aligned}$$

The FI of $\delta$ from a single measurement (a single choice of $t$) is given by

4 $$\begin{aligned} j_{\delta ,\delta } \approx \frac{c^2}{4 \eta +c^2}\phi _{\text {rms}}^4 t^2 \sin ^2 (\delta t ) C^2( t/T_\phi ) , \end{aligned}$$

in the weak signal regime. Eq. (4) shows that the sine term is the reason for the limited resolution. The maximum amount of information from a single measurement (for small $\delta$) depends on the correlation function. An exponential decay imposes an optimal measurement time that scales as $t^\text {opt}\propto T_\phi$; i.e., the longest time possible before the correlation is exponentially small. Thus the information scales as $j_{\delta ,\delta } \propto \delta ^2 T_\phi ^4$, and vanishes for $\delta \rightarrow 0$. By contrast, for a slow polynomial decay (i.e., $C(z) \propto z^{-n}$ for large $z$ and $0.5<n<1.5$, with *z* henceforth being $z = t/T_{\phi }$) the optimal measurement time scales as $t^\text {opt}\propto \delta ^{-1}$; i.e., the correlations are significant enough such that the sine term poses no problems. Thus the information scales as $j_{\delta ,\delta } \propto \delta ^{2n-2}T_\phi ^{2n}$, with a weaker dependence on frequency. With respect to the measurement time, the information rate is $j_{\delta ,\delta }/T_\text {tot} \propto \delta ^{2n-1}T_\phi ^{2n}$; consequently, for correlations with $n<1.5$ there is a slight improvement in resolution, and for $n=1.5$, as in^17^ (Eq. 13), there is no improvement over exponential correlations. For this reason it may be desirable to consider different measurement protocols.

#### Qdyne/synchronized measurements

Further improvement can be made considering a synchronized measurement protocol^1,8,9^. In this scenario, the fluorescence response of the NV has a detection rate of

5 $$\begin{aligned} q_t = \eta + \frac{c}{2} \sin (\phi _t ). \end{aligned}$$

Thus the average probability for measuring the pair $(y_s,y_{s+t} )$ of number of photons is

6 $$\begin{aligned} \langle q_s q_{s+t}\rangle = \eta ^2 + \frac{c^2}{4} e^{-\phi _\text {rms}^2} \sinh \big (\phi _\text {rms}^2 \cos ( \delta t ) C (t/T_\phi ) \big ). \end{aligned}$$

Estimating the signal using the covariance between the number of photons detected at different times, the information about $\delta$ (from two measurements with a time difference $t$) is given by

7 $$\begin{aligned} j_{\delta ,\delta } = \frac{c^4}{(4 \eta +c^2)^2} \phi _{\mathrm{rms}}^4 t^2 \sin ^2 (\delta t ) C^2( t/T_\phi ) + \mathscr {O}(\phi _{\mathrm{rms}}^6 ). \end{aligned}$$

**Figure 2:**
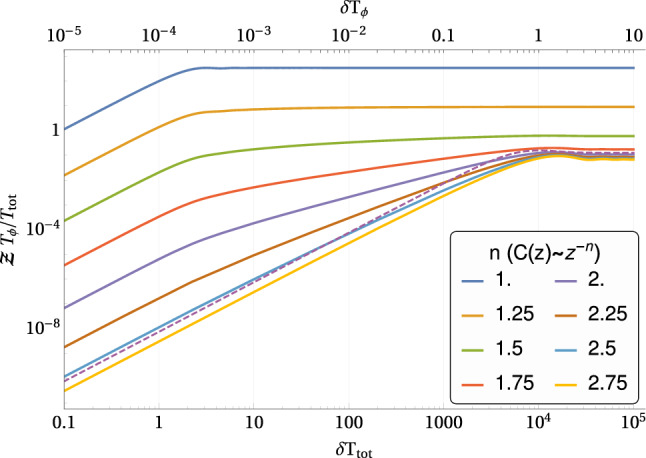
Scaling of the FI rate about $\delta$ as a function of $\delta$ (Eqs. 8, 9); for this plot we set $T_\text {tot}=10^4 T_\phi$. Different polynomial scalings are presented in different colors. The case of exponential correlation is presented as a dashed line. The information per unit of time saturates for $\delta T_\text {tot} \gtrsim 1$, for correlations with slow polynomial decay ($n<1.5$). For faster decays ($1.5<n<2.5$) the characteristic time changes continuously towards $\delta T_\phi \gtrsim 1$ (see top horizontal axis). For the limiting case of $n=1.5$ the information rate changes its behavior for $\delta T_\text {tot} \gtrsim 1$, but only saturates for $\delta T_\phi \gtrsim 1$, which is attributed to the small logarithmic correction $\log (\delta T_\phi )$.

This FI is obtained for a weak signal by (least-squares) fitting of the correlation function. With each additional measurement (performed at time $t+\tilde{\tau }$) we effectively obtain $t/\tilde{\tau }$ additional “measurements” by correlating with all previous measurements. For small rms we can safely assume that the noise in the “measurements” is uncorrelated. For data taken at times $t_m = m\tilde{\tau }$, the total FI is given by

8 $$\begin{aligned} J_{\delta ,\delta }&\approx \frac{c^4}{(4 \eta +c^2)^2}\phi _\text {rms}^4 \frac{T_\phi ^4}{\tilde{\tau }^2} \mathscr {Z} , \end{aligned}$$

9 $$\begin{aligned} \mathscr {Z}&= \intop _0^{T_\text {tot}/T_\phi } z^2 \sin ^2 (\delta T_\phi z ) C^2(z) \left( \frac{T_\text {tot}}{T_\phi }-z \right) \text {d}z , \end{aligned}$$

where we assumed $\delta \tilde{\tau }$ and $\tilde{\tau }/T_\phi$ to be small. The behavior of the integral in Eq. (9) for small $\delta$ depends on the correlation function. For an exponential decay, $\mathscr {Z}\propto \delta ^2 T_\phi T_\text {tot}$ in the regime of $\delta T_\phi \ll 1 \ll \delta T_\text {tot}$, whereas for polynomial decay

10 $$\begin{aligned} \mathscr {Z}\propto {\left\{ \begin{array}{ll} ( {T_\text {tot}}/{T_\phi })^{4-2n}&{}, n<1.5 \\ {\delta T_\text {tot}} (\delta T_\phi )^{2n-4}&{}, 1.5<n<2.5 \\ \delta ^2 T_\phi T_\text {tot} &{}, n > 2.5 \end{array}\right. } \end{aligned}$$

in other words, there is a minute correction for small $\delta$ when the polynomial decay is slower than $2.5$. For decay rates slower than $1.5$ the information is independent of $\delta$, and the information rate increases with time ($\propto T_\text {tot}^{3-2n}$) (see Fig. 2). In the limiting case of $n=1.5$, $\mathscr {Z} \propto \log ( \delta T_\text {tot} ) T_\text {tot}/T_\phi$ and the correction grows logarithmically when $T_\text {tot}$ is large.

Compared to the correlation spectroscopy in Eq. (4), the information from synchronized measurements in Eq. (7) suffers from an extra $c^2/(4\eta +c^2)$ factor (which is small in current experiments) due to correlations being obtained at post-processing rather than on the NV. Nevertheless, this factor is compensated for by the fact that more statistics are gathered in Qdyne; i.e., roughly a factor of $(T_\text {max}/\tilde{\tau })^2$, assuming correlation spectroscopy measurements are performed using sequantial correlation times up to time $T_\text {max}$. For exponential decays $T_\text {max} \sim T_\phi$ and $T_\text {max} \sim \delta ^{-1}$ for slow polynomial decays, as seen in Eq. (4). These extra statistics compensates the logarithmic correction for small $\delta$, meaning that the resolution with Qdyne is not limited by $T_\phi ^{-1}$.

Note that for correlation spectroscopy the shortest correlation time is limited by the DD sequence (which must be shorter than the coherence time of the signal), whereas for Qdyne is limited also by the readout/initialization time ($\tilde{\tau }-\tau \approx 2.1 \mu s$, see for example^8^); for exponential correlations this limits the Qdyne technique to samples with coherence time longer than the readout time. But for a slow polynomial decay this induces only a small constant factor on the information, as most of the information comes from long-time correlations.

#### Power spectrum measurements

In the power spectrum measurement scenario, the interrogation time, $\tau$, must be increased beyond the correlation time of the noise, which in most cases is impossible since the coherence time of the NV ($T_2^{\text {NV}}$) is too short. The fluorescence response of the NV is given by

11 $$\begin{aligned} \langle y_\omega \rangle = \eta - \frac{c}{2} \exp \left( -\frac{1}{2} \gamma ^2 B_\text {rms}^2T_\phi \tau \mathscr {S}_\tau (\omega )\right) , \end{aligned}$$

where $\mathscr {S}_\tau (\omega )$ is the unit-less (normalized by $T_\phi \tau$) power spectrum (convoluted with the filter function defined by the DD protocol). The restriction on the interrogation time poses an extra limit on the field strength being probed $\gamma ^2 B_\text {rms}^2T_\phi \tau \lesssim 1$ (i.e., a large rms value will saturate the signal exponentially fast). In addition, the inverse interrogation time sets the resolution for this measurement protocol; i.e., in order to resolve a frequency difference $\delta$ we must set $\tau > \delta ^{-1}$.

**Figure 3:**
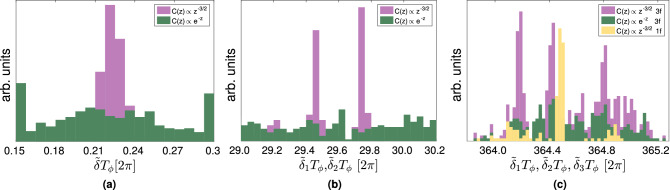
**(a)** One frequency below the Rayleigh Limit is estimated for correlation $C(z\gg 1)\propto z^{-3/2}$ (purple) whereas estimation is not possible for exponential decay (green). $\phi _\text {rms}$ of the signal is 0.6. In purple, combinations of 50 estimation instances for each of the 12 different NV depths normalized to $\hbox {T}_\phi$. Signal noise in this case is generated by randomly taking vectors of length N from MD data (see “Methods”). In green, result for signals with the same parameters but with noise which is generated by fitting MD data to an exponential and fitting the signal to Eq. (12) with $C(z\gg 1)\propto z^{-3/2}$. **(b)** Two frequencies with a frequency difference ($\Delta \delta )T_\phi = 0.3 [2\pi ]$ are resolved for long-lived correlations (purple) but remain unresolved for exponential decay (green). The amplitude of the signal is $\phi _\text {rms} \approx$ 0.6. Each histogram contains correlation function fittings of 200 measurement vectors with $2^{14}$ measurements. **(c)** Three frequencies (purple) with a frequency separation below the Rayleigh Limit, $(\Delta \delta ) T_\phi \approx$ 0.3 [$2\pi$], are resolved for the case of long-lived correlations $C(z\gg 1)\propto z^{-3/2}$. For exponentially decaying correlations the same signal produces a histogram in which no single frequency can be pinpointed. In yellow, we generate a single-frequency signal. A signal with one frequency is estimated showing that the MSE is commensurate with the multi-frequency analysis.

When these requirements are met, the shape of the spectrum will dictate the information scaling; correlations that decay with a power law $-n$ correspond to a spectrum that scales with a power law $n-1$ around the peak. For a smooth spectrum ($n>2$) the information scales as the derivative of the spectrum (squared), $j_{\delta ,\delta } \propto T_\phi ^2 (\delta T_\phi )^{\text {min}[2n-4,2]}$ at $\omega =0$. For a sharp spectrum (derivative is discontinuous at the peaks, $1<n<2$) the optimal measurement is performed at $\omega -\delta \propto \tau ^{-1}$ (as close as possible to the peak, before the shape of the filter function starts to dominate) and the information scales as $j_{\delta ,\delta } \propto T_\phi ^2 (\tau / T_\phi )^{4-2n}$. For the former case, resolution limit is set by $T_\phi ^{-1}$, albeit with a reduced “penalty”, and by $\tau ^{-1}$ for the latter.

### Nano-NMR signal analysis

We now demonstrate resolution and verify the theoretical analysis by simulating and analyzing both single and multi-frequency signals. The procedure is as follows; First, we generate accumulated phases $\phi _t$ (Eq. 5) by either using molecular dynamic (MD) simulations for a more accurate description of an experimental situation (see “Methods”), or we sample a multivariate Gaussian distribution which simplifies the theoretical analysis. These phases are then used to simulate measurement vectors in a Qdyne protocol. Parameter estimation is then performed by least squares fitting the signal correlation function to the theoretical model

12 $$\begin{aligned} \sum _i (\phi _\text {rms}^{(i)})^2 \cos (\delta _i t + \varphi _i)C(t/T_\phi ), \end{aligned}$$

which corresponds to Eq. (6) for weak signals. $C( z )$ is considered either as polynomial correlations $\propto z^{-3/2}$ corresponding to Eq. (13) from^17^ (henceforth $C(z\gg 1)\propto z^{-3/2}$), or an exponential correlation $\exp (-z)$ for comparison purposes. The $\varphi _i$ in Eq. (12) is a dummy parameter added for numerical reasons, and which tends to zero. For more information about the numerical procedure see “Methods”.

#### Resolution

Figure 3 illustrates resolution beyond the Rayleigh Limit. We generate the signals of the magnetic field at different NV depths by using MD simulations of $\hbox {N} \approx 46\hbox {k}$ dipolar particles diffusing as a Lennard-Jones fluid, whose correlations behave as $C(z\gg 1)\propto z^{-3/2}$ at long times. Comparison to an exponential correlation function decay is done by fitting the MD results to an exponential model and using this model as a noise source. In generating the signals, each NV-depth from MD is used, and is appropriately scaled according to the $\hbox {T}_{\phi }$ associated with the NV depth at which it is measured. Moreover, we work in the limit of $\delta T_\phi$ small ($\sim 0.3 [2 \pi ]$) and small $\phi _{\mathrm{rms}}$ ($\sim 0.6$), where as in the theoretical analysis shown in Eq. (9) the exponential correlations limit the resolution.

In Fig. 3a we depict the estimation of a single frequency for 600 measurement vectors, each composed of $2^{12}$ measurements. In fitting the correlation function Eq. (12), a fitting is only accepted if $r^2>$ 0.95. Fig. 3b depicts resolution for two close frequencies, which in this case loosely correspond to those of the experiment in^16^ but performed with an applied magnetic field one order of magnitude smaller. For this case we generate 200 measurement vectors of $2^{14}$ measurements each. A fitting is accepted if $r^2 > 0.95$. In both cases, the frequencies were not resolved for the same parameters but rather with exponential correlations.

Estimating close frequencies is a global optimization problem whose complexity increases exponentially in parallel with the size of the search space in which the frequencies live. In Fig. 3c we depict the resolution of three close frequencies which correspond to the frequencies from the experiment by Glenn et al.^1^ but performed with a non-polarized sample. This is compared to a signal generated with exponential correlations, which does not allow for resolution of the frequencies. Furthermore, we include the histogram corresponding to a signal with one frequency slightly offset from the central frequency of^1^, generated with the same parameters and analyzed in the same way. It demonstrates that the Mean Square Error (MSE) is independent of the number of frequencies.

#### Scaling analysis

**Figure 4:**
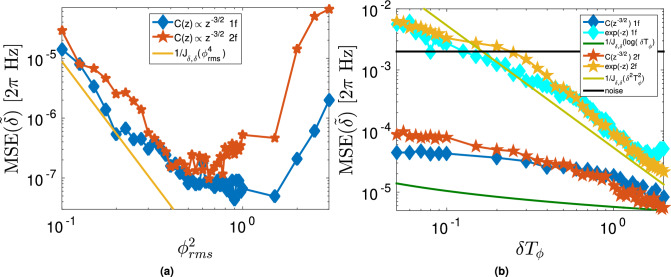
**(a)** MSE of the frequency estimator $\tilde{\delta }$ (blue) and frequency difference estimator $\Delta \tilde{\delta }=|\tilde{\delta }_1 - \tilde{\delta }_2|$ (orange) as a function of $\phi _\text {rms}$ with $\delta T_\phi = 0.5 [2\pi ]$. The line shows the theoretical prediction from Eq. (8) (valid only for small $\phi _{\mathrm{rms}}$) dominated by $1/\phi _\text {rms}^4$. Below $\phi _\text {rms} \approx$ 0.1 the MSE saturates, indicating that the estimator is distributed across the whole search region. **(b)** MSE of the frequency estimator $\tilde{\delta }$ and frequency difference estimator $\Delta \tilde{\delta }=|\tilde{\delta }_1 - \tilde{\delta }_2|$ for fixed $\phi _\text {rms} = 0.6$ as a function of $\delta T_\phi$ for polynomial $C(z\gg 1)\propto z^{-3/2}$ (diamonds) and exponential (stars) correlations. Horizontal line in **(b)** represents the flat histogram limit (noise level). Solid lines are the theoretical predictions from Eq. (10) for $\hbox {n} = 1.5$ (dark green) proportional to $1/\log (\delta T_{tot})$, and exponential (light green) $\propto 1/(\delta T_\phi )^2$. Each point represents the MSE of $2^8$ measurement vectors with $2^{14}$ measurements per vector. Note that the small differences between the one frequency and two frequency cases are merely numerical artifacts which would diminish for a higher number of measurement vectors. In both plots $\hbox {T}_{\text {tot}}/T_\phi \approx 164$ for all points. In **(a)** $\delta T_\text {tot} \approx 82 [2\pi ]$.

We now proceed to the numerical analysis of the theoretical model presented in the previous section, in the case of one and two frequency signals. We show that for the anticipated signal in the nano-NMR scenario, the characteristic time for resolution is the total measurement time. In this case, we simulate synchronized measurements by generating signals with an analytical correlation function $C(t/T_\phi )$ where the noise comes from sampling a multivariate Gaussian distribution mimicking the scenario of small $\phi _\text {rms}$. We focus here on the case of n = 1.5 in Eq. (10) corresponding to the correlation function in Eq. (13) ($C(z\gg 1)\propto z^{-3/2}$) from^17^. A point in Fig. 4 corresponds to the MSE of a histogram composed of $\hbox {N} = 2^8$ measurement vectors each, with $2^{14}$ measurements.

Figure 4a displays the behavior of the MSE of the estimator as a function of $\phi _\text {rms}$. For fixed $\delta T_\phi = 0.5 [2\pi ]$, below the Rayleigh Limit such that the signal with an exponential correlation could not be resolved, we simulate signals with varying $\phi _\text {rms}$. According to Eq. (10), for a weak signal the MSE (i.e. 1/$J_{\delta ,\delta }$) diverges as $\phi _{\mathrm{rms}}^{-4}$ as we observe in Fig. 4b, thus setting the optimal region for nano-NMR around $\phi _\text {rms} = 1$. For strong signals, the information rate is exponentially suppressed. The scaling in the case of one frequency is not fundamentally different from that of two frequencies.

In Fig. 4b we set $\phi _{\text {rms}} = 0.6$ and study the behavior with $\delta T_\phi$. Here we can observe the difference caused by extended correlations in the information rate and thus the resolution capacity. While for exponential correlations the MSE diverges quadratically with $\delta$, and rapidly saturates the histogram, for polynomial decays the divergence is slower. In the case of $C(z\gg 1)\propto z^{-3/2}$ the divergence is logarithmic in $\delta$ (see Eq. (10)), as we see in Fig. 4b, i.e., it can easily be compensated for by increasing the measurement time. Note in addition that since $\phi _{\mathrm{rms}}$ ($B_{\mathrm{rms}}$) $\sim 1/d^{3/2}$ and $T_\phi \sim d^2$^15^, for $C(z\gg 1)\propto z^{-3/2}$ according to Eq. (10) the MSE is independent of the depth of the NV, as occurs with polarized nano-NMR.

## Discussion

We showed that spectral resolution in non-polarized liquid state nano-NMR is not necessarily limited by the broadening of spectral lines due to diffusion. While for exponential correlations the resolution is limited by the inverse characteristic coherence time of the signal, we demonstrate that for (slow) polynomial correlations, as predicted by^17^, resolution is not limited.

We analyzed the scenario in which the sensor is a shallow NV center. In this case, the correlations decay as $C(z\gg 1)\propto z^{-3/2}$ at long times, producing sharp spectral features. Moreover, increasing the number of frequencies analyzed does not hinder resolution.

Comparing the three measurement protocols we observe that for exponential correlations, the resolution problem always appears for $\delta T_\phi < 1$, but the sensitivity of Qdyne is different by a factor of about $(c^2/\eta )(T_\phi /\tilde{\tau })^2$. For a low viscosity, water-like fluid this could still prove beneficial, despite the low contrast in state of the art systems ($c^2/\eta \approx 0.016$). For power-law decay (with power of 3/2), while the sensitivity remains the same as the exponential case, the resolution capabilities of the power spectrum measurement and Qdyne protocols are extended. For power spectrum measurements, the protocol is limited by the time of a single measurement ($\tau$) which is only restricted by the coherence time $T_2$ of the NV sensor. The Qdyne protocol is virtually not limited by diffusion as the only limitation is the total measurement time.

The power law analysis presented here is so far based on theoretical grounds. Nonetheless, experimental evidence for a deviation from the exponential correlations paradigm already exist. In fact, Staudacher et al. found in^20^ a correlation function for a non-polarized liquid state nano-NMR experiment which exhibits a long-lived tail. Such behaviour was attributed to a surface effect which creates a thin layer of static, rotating molecules close to the surface of the diamond, finding a reasonably good agreement between the model and the experimental results. It is clear that the assumption of macroscopic Brownian motion with a Lorentzian profile and exponential correlations is too crude an approach to the non-polarized nano-NMR setting. As such, the diffusion induced long-lived correlations described in^17^, which we have demonstrated lead to enhanced resolution, are but a lower limit on the achievable resolution scaling of the non-polarized nano-NMR setup. Different physical effects such as those described in^20^ demonstrate that even longer-lived correlations can be expected to exist. As our analysis demonstrates, harnessing these power-law correlations leads to an increase of the information gathered (see Fig. 2), resulting in even better scaling for resolution of frequencies in a nano-NMR spectra.

## Methods

### Noise model for diffusing particles

Each nucleus composing the sample substance interacts with the NV center via dipolar coupling; in the nano-NMR setting, nucleus dynamics manifests through the dephasing rate of the NV center. Calculating this dephasing rate involves solving the drift-diffusion dynamics equation. For an NV situated at a depth *d* from the diamond surface and assuming that the liquid fills a semi-infinite volume above the diamond surface, the correlation function for the nucleus distribution is^17^

13 $$\begin{aligned} C(z) = \frac{4}{\sqrt{\pi }} \Bigg ( z^{-\frac{3}{2}} - \frac{3}{2}z^{-\frac{1}{2}} + \frac{\sqrt{\pi }}{4} + 3\sqrt{z} - \frac{3\sqrt{\pi }}{2}z + \sqrt{\frac{\pi }{z}}{{\,\mathrm{erfc}\,}}\Big (z^{-\frac{1}{2}}\Big )\exp {z^{-1}}\Big ( - z^{-\frac{3}{2}} + z^{-\frac{1}{2}} - \frac{7}{4}\sqrt{z} + \frac{3}{2}z^{+\frac{3}{2}} \Big ) \Bigg ), \end{aligned}$$

with $z = d^{-2} D t = {t}/{T_\phi }$, where *D* is the diffusion coefficient for the fluid.

To accurately simulate the NV response signal to the magnetic field generated by a distribution of diffusing molecules used to demonstrate resolution in an experimental-like scenario, we perform molecular dynamics simulations.

For the molecular dynamics we consider $N\approx 46k$ dipolar particles within a simulation box of size $L_{x,y,z} \approx (50,50,24)$, with a NV located at depths in the range of (0.3, 5). The particles within the box are simulated as a Lennard–Jones fluid with normalized parameters $\varepsilon =\sigma =1$, and are initialized into a thermal state at temperature $T=1$. During the simulation, the magnetic field induced by the particles at the NV position is measured along the *z* direction for several NV depths.

**Figure 5:**
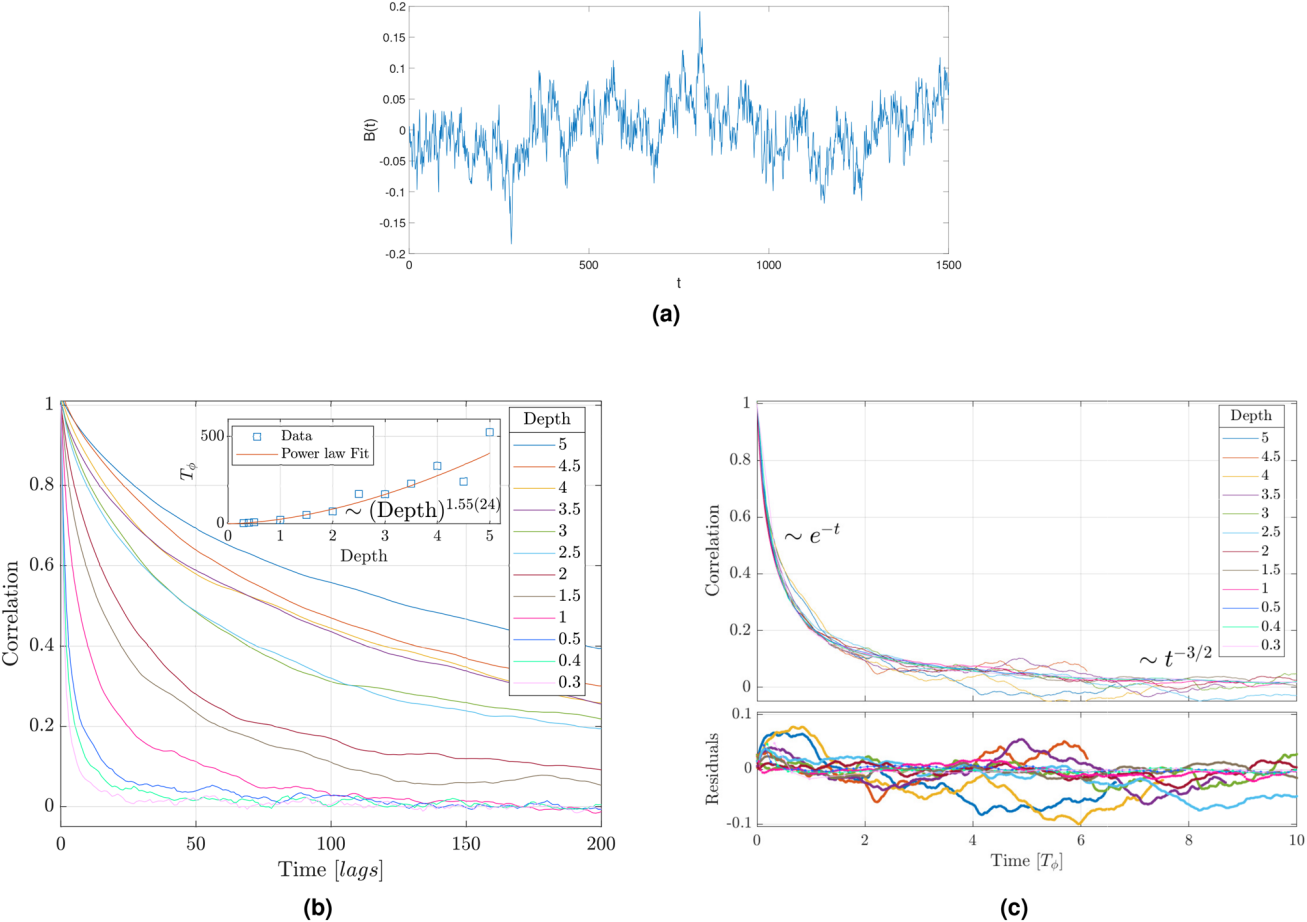
**(a)** Sample of the molecular dynamics results for the magnetic field created at the NV position by a distribution of randomly diffusing dipolar particles. **(b)** Correlation function of the magnetic field created at the NV position for depths ranging from 0.3 to 5. Each correlation curve is the average of two realizations of molecular dynamics. The inset shows the correlation time of the magnetic field as a function of depth, obtained by fitting the correlation data to a correlation $C(z\gg 1)\propto z^{-3/2}$ (FT of Eq. 13). Each $T_\phi$ is calculated as $C(z = {t}/{T_\phi } \approx 0.255791) = {1}/{2}$. Deviations from the theoretical exponent ($T_\phi \propto d^2$) occur due to finite box-size and simulation errors. Shallower NVs feature a different box-size; hence, a departure from a straight line. **(c)** Correlation of the magnetic field scaled to $T_\phi$. At short-times the correlation decays exponentially, whereas at long-times the decay is polynomial. This demonstrates that the diffusing particles create a highly correlated signal. Residuals are with respect to fitting in Fig. 5a.

Analysis of the generated magnetic fields at different NV depths shows that the data have no trend and that the standard deviation remains scale invariant. This means we can compare different depths if appropriately scaled. This is done by calculating the correlations and partial correlations of the different time series. An example of a time series can be found in Fig. 5a.

In Fig. 5b we analyze the temporal correlation in the magnetic field as a function of NV depth. The correlation, which is akin to the autocorrelation after correcting by the mean, tells us how a point in the time series is related to itself after k time-steps. We observe that it is highly dependent on the depth of the NV, as expected from the relation $T_\phi \propto d^2$ (the diffusion coefficient D is the same for all depths). Since resolution depends on $\delta T_\phi$ Fig. 5b gives us information about which depth is more convenient, depending on the characteristics of the signal that we want to analyze.

Figure 5c depicts the correlation corrected by depth. Note the deviation from exponential decay at long times, as described by Eq. (13), which is responsible for long-lived correlations. Moreover, this deviation is independent of the depth of the NV, which means that the same description is valid for the magnetic field at any NV depth.

When using MD vectors to simulate the accumulated phases $\phi _t$, we avoid correlations among different MD vectors by calculating each noise realization by randomly sampling two different instances of magnetic fields in the corresponding NV depth.

### Numerical calculations

Parameter estimation is done by numerically fitting each measurement vector to the theoretical model

14 $$\begin{aligned} \sum _i (\phi _\text {rms}^{(i)})^2 \cos (\delta _i t + \varphi _i)C(t/T_\phi ). \end{aligned}$$

The fitting is done by a non-linear least squares algorithm with finite-difference estimation of gradient. Each fitting is initialized with random values taken from uniform distributions around the mean signal values for each parameter in Eq. (12). The width of the distributions coincides as well with the allowed search regions in the fitting process. These are, respectively, $\phi _{\text {rms}} \in$ [$\phi _{\text {rms}}^{\text {avg}}/2, 3\phi _{\text {rms}}^{\text {avg}}/2$], $\delta \in$ [$\delta ^{\text {avg}}/2, 3\delta ^{\text {avg}}/2$], $\varphi \in$ [$0, 2\pi$] and $T_\phi \in [T_\phi /100, 100T_\phi ]$. Average values are estimated from the signal for the $\phi _{\text {rms}}$ or from the signal FT for $\delta$.

The $\varphi _i$ in Eq. (14) is non-physical and is included for reasons of numerical stability. In all of the fittings it tends to either 0 or $2\pi$.

## Supplementary information

Supplementary Information.
