# Supplementary material for: Correlated noise in Brownian motion allows for super resolution
Source: Sci Rep. 2020 Nov 12;10:19691. doi: 10.1038/s41598-020-76745-4 (PMC7661544; doi:10.1038/s41598-020-76745-4)
Supplement: Supplementary file 1 — Supplementary Information. [file 41598_2020_76745_MOESM1_ESM.pdf]

Correlated noise in Brownian motion allows for super resolution. Supplementary Information.

Santiago Oviedo-Casado[1,ℑ,℘], Amit Rotem[1,ℑ,ℵ], Ramil Nigmatullin[2], Javier Prior[3,4], and Alex Retzker[1]

[1]Racah Institute of Physics, The Hebrew University of Jerusalem, Jerusalem 91904, Givat Ram, Israel
[2]Complex Systems Research Group and Centre for Complex Systems, Faculty of Engineering and IT, The University of Sydney, Sydney, NSW 2006, Australia.
[3]Área de Física Aplicada, Universidad Politécnica de Cartagena, Cartagena 30202, Spain
[4]Instituto Carlos I de Física Teórica y Computacional, Universidad de Granada, Granada 18071, Spain
[ℑ]these authors contributed equally
[℘]oviedo.cs@mail.huji.ac.il
[ℵ]amit.rotem1@mail.huji.ac.il

1 Gaussian noise model

We consider a two level system (TLS) coupled to a control field and a noisy field, in the interaction picture with respect to the TLS energy gap. We describe it as the Hamiltonian

$$\mathscr{H} = \Omega(t)\sigma_\zeta + \frac{\gamma}{2}B(t)\sigma_z, \tag{S1}$$

where $\Omega(t)$ represents the controls being applied to the sensor (ζ is perpendicular to z). The second term is the noise signal that we want to measure, with γ the coupling constant of the field. We assume that the field B is a stationary Gaussian process with mean zero and covariance

$$\mathrm{cov}(B_s, B_{s+t}) = B_{\mathrm{rms}}^2 \mathscr{C}(t), \tag{S2}$$

where B_{rms}^2 is the variance of the field, and $\mathscr{C}(\cdot)$ is the normalized covariance function.

Following the derivation by Cywińsky et al.[1], we consider the control field as a sequence of (infinitely fast) n π pulses at times $\{t_j\}_{j=1}^n$; e.g., for a CPMG sequence $t_j = (j - \frac{n+1}{2})\tau/n$, about an axis that is perpendicular to z. We define $t_0 = -\tau/2$ ($t_{n+1} = \tau/2$) as the start (end) of the sequence. The Hamiltonian in the interaction picture with respect to these pulses is given by

$$\mathscr{H}_I = h(t)\frac{\gamma}{2}B(t)\sigma_z, \tag{S3}$$

where $h(t)$ is the response function, which for times $t_j < t < t_{j+1}$ is equal to $+1$ (-1) for even (odd) j and zero for $t < t_0$ or $t > \tau$; e.g., for CPMG $h(t) = \theta(t+\tau/2)\theta(\tau/2-t)\mathrm{sgn}(\cos((t-\tau/2)n\pi/\tau))$, where $\theta(\cdot)$ is the Heaviside step function, and $\mathrm{sgn}(\cdot)$ is the sign function. The accumulated phase on the qubit

$$\phi(t) = \gamma \int_{-\tau/2}^{\tau/2} \mathrm{d}a\, h(a)B(t+a), \tag{S4}$$

is a stationary Gaussian process with mean zero and covariance

$$\mathrm{cov}(\phi_s, \phi_{s+t}) = \gamma^2 B_{\mathrm{rms}}^2 \int_{-\tau/2}^{\tau/2} \mathrm{d}a \int_{-\tau/2}^{\tau/2} \mathrm{d}b\, \mathscr{C}(t+b-a)h(a)h(b) \tag{S5}$$

$$= \gamma^2 B_{\mathrm{rms}}^2 \int_{-\infty}^{\infty} \mathrm{d}f\, \mathscr{S}(f)F(f)e^{i2\pi ft}, \tag{S6}$$

where $\mathscr{S}(f)=\int_{-\infty}^{\infty}\mathscr{C}(t)e^{-i2\pi ft}\mathrm{d}t$ is the power spectrum of $B(t)$. $F(f)=\left|\widetilde{h}(f)\right|^2$ is the filter function which is defined by the pulse sequence $\widetilde{h}(f)=\int_{-\tau/2}^{\tau/2}h(t)e^{-i2\pi ft}\mathrm{d}t$; e.g., for CPMG

$$F(f)=\frac{4}{\pi^2 f^2}\frac{\sin^2\left(\pi f\tau+\frac{n\pi}{2}\right)\sin^4\left(\frac{\pi f\tau}{2n}\right)}{\cos^2\left(\frac{\pi f\tau}{n}\right)} \tag{S7}$$

$$=\left|\frac{2\tau i^n}{\pi}\sum_{m=-\infty}^{\infty}\frac{(-1)^{(n+1)m}}{1+2m}\mathrm{sinc}\left(\left(f-(1+2m)\frac{n}{2\tau}\right)\pi\tau\right)\right|^2 \tag{S8}$$

$$\approx\frac{4\tau^2}{\pi^2}\left(\mathrm{sinc}\left(\left(f-\frac{n}{2\tau}\right)\pi\tau\right)+(-1)^n\mathrm{sinc}\left(\left(f+\frac{n}{2\tau}\right)\pi\tau\right)\right)^2. \tag{S9}$$

The main peaks of this function are located at $f=\pm n/(2\tau)+\mathscr{O}(n^{-1})$, with a full width half max of about $1/\tau$, and an area of $4\tau/\pi^2$ each.

Eq. S6 has two regimes of interest to this manuscript. When the width of the filter function (τ^{-1}) is smaller than the bandwidth of the signal (W), and when filter function is wider. They are denoted as

$$\mathrm{cov}(\phi_s,\phi_{s+t})\propto\gamma^2B_{\mathrm{rms}}^2\begin{cases}\tau\mathscr{S}(f_{\mathrm{DD}}) & ,\tau^{-1}\ll W\\ \tau^2\mathscr{C}(t) & ,\tau^{-1}\gg W\end{cases} \tag{S10}$$

where $f_{\mathrm{DD}}=n/(2\tau)$ is the probing frequency (DD frequency). The former regime is appropriate for direct spectrum measurement, and the latter for correlation spectroscopy and synchronized measurement protocols. The following sections analyze the problem of resolution in these regimes.

2 Resolution problem

Resolution is defined as the ability to differentiate between close frequencies. To explore the resolution problem we focus on a simplified scenario where the signal ($B(t)$) is a narrow band noise and is composed of only two frequencies; i.e., $B(t)=\sum_{k=1}^{2}a_k(t)\cos(\omega_k t)+b_k(t)\sin(\omega_k t)$ where each $\{a_k,b_k\}$ are stationary Gaussian processes with spectrum ($S_k(f)$) centered around $f=0$. In this case the resolution problem emerges because of symmetries in the labeling (k) of the frequencies (i.e., $1\leftrightarrow 2$)[2], when the likelihood that the set of parameters (θ_1,θ_2) that created the measurements set overlaps with the likelihood that the set (θ_2,θ_1) created the same measurements. Here θ_k denotes the set of parameters that characterize the process $\{a_k,b_k\}$ (or equivalently the spectrum) and the frequency ω_k. We focus on the special case in which the processes $\{a_k,b_k\}$ all have the same autocorrelation, and are all characterized by some coherence time T_ϕ and signal strength B_{rms}. Consequently the symmetries only affect the swapping of frequencies; i.e., $\omega_1\leftrightarrow\omega_2$. Generally speaking, the central frequency is easier to estimate[2], so we reduce the problem further to that of estimating a single frequency (δ) that is closer to zero as compared to the noise band-width ($\approx T_\phi^{-1}$); i.e., $\delta\ll T_\phi^{-1}$.

We denote the general form of the signal considered in the rest of this manuscript

$$B(t)=a(t)\cos(\delta t)+b(t)\sin(\delta t), \tag{S11}$$

$$\mathrm{cov}(B_s,B_{s+t})=\frac{4}{\pi^2}B_{\mathrm{rms}}^2\cos(\delta t)C(t/T_\phi), \tag{S12}$$

and $C(z)$ is either $e^{-|z|}$ or

$$\begin{cases}e^{-|z|} & ,|z|\le 1\\ e^{-1}|z|^{-n} & ,|z|>1\end{cases},$$

as an approximation for

$$C(z)=\frac{4}{\sqrt{\pi}}\left(z^{-\frac{3}{2}}-\frac{3}{2}z^{-\frac{1}{2}}+\frac{\sqrt{\pi}}{4}+3\sqrt{z}-\frac{3\sqrt{\pi}}{2}z+\sqrt{\frac{\pi}{z}}\,\mathrm{erfc}\left(z^{-\frac{1}{2}}\right)\exp z^{-1}\left(-z^{-\frac{3}{2}}+z^{-\frac{1}{2}}-\frac{7}{4}\sqrt{z}+\frac{3}{2}z^{+\frac{3}{2}}\right)\right), \tag{S13}$$

as calculated in[3].

For short interrogation times $\tau \ll T_\phi$ the response function simplifies to

$$h(t) = \theta(t+\tau/2)\theta(\tau/2-t), \tag{S14}$$

and the covariance Eq. S5 can be written as

$$\mathrm{cov}(\phi_s, \phi_{s+t}) = \tau^2 \mathrm{sinc}^2(\delta\tau/2)\mathrm{cov}(B_s, B_{s+t}). \tag{S15}$$

3 Problem illustration in the spectrum

The line shape for a polynomial correlation of power $(-n)$ with $n < 3$, behaves as $S(f) \approx 1 - \alpha|f|^{n-1}$ around the peak. For a noisy signal containing two frequencies the spectrum is given by $\mathscr{S} \approx S(f+\Delta f) + S(f-\Delta f)$, and the derivative with respect to the frequency difference scales as Δf^{n-2}. On the opposite end, for a Gaussian or Lorentzian line shape we have that $S(f) \approx 1 - \alpha f^2$, and the derivative goes to zero as the frequencies overlap. This means that the diffusion process responsible for polynomial correlations as explained in Cohen et al.[3] does not limit the spectral resolution, as occurs in conventional NMR. In practice, other factors will limit the resolution, such as the measurement time (a single interrogation time) in power spectrum measurements. In what follows we analyze resolution in terms of these factors.

4 Correlation spectroscopy

We consider the following measurement protocol;

$$\begin{aligned}
&\text{Initialize the NV to its ground state,}\\
&\text{pulses: } \mathrm{R}_y(\pi/2) - \mathrm{DD}(f_{\mathrm{DD}}, \tau) - \mathrm{R}_x(\pi/2),\\
&\text{wait } (t-\tau),\\
&\text{pulses: } \mathrm{R}_y(\pi/2) - \mathrm{DD}(f_{\mathrm{DD}}, \tau) - \mathrm{R}_{-x}(\pi/2),\\
&\text{state readout,}
\end{aligned} \tag{S16}$$

where $\mathrm{R}_u(\theta)$ is a rotation of angle θ around the u axis. $\mathrm{DD}(f_{\mathrm{DD}}, \tau)$ stands for some dynamical decoupling sequence at frequency f_{DD} with total duration τ. The dynamics during the DD sequences are given by the Hamiltonian in Eq. S1. We assume that a T_2 dephasing process erases the phase information during the wait time, but does not affect the state during the DD pulses sequence; meaning that the correlation time t is limited by $T_1^{(NV)}$, and that the DD sequence time τ is limited by T_2.

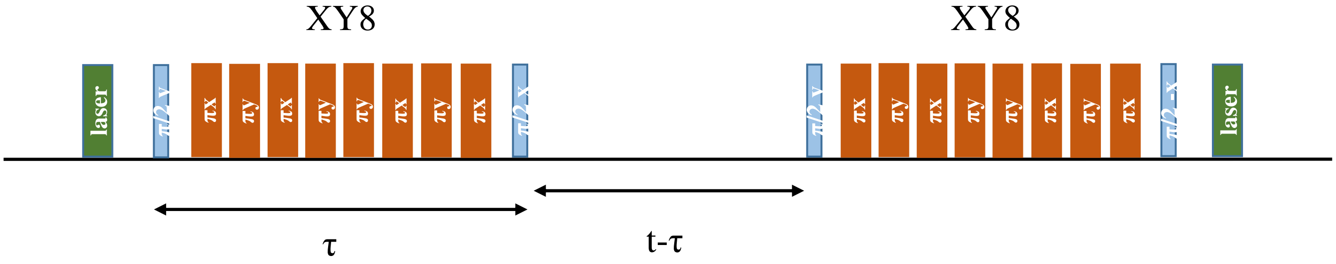

Figure S1. Correlation spectroscopy measurement protocol. Following initialization of the NV via a 532 nm laser, a dynamical decoupling sequence gathers information about the sample and stores it the population of the NV. Following an erasure time, a second dynamical decoupling sequence gathers a second phase which then is correlated with the first one upon state readout.

The probability of the NV to be in the excited state is

$$p_{s,t} = \frac{1}{2} + \frac{1}{2}\sin(\phi_s)\sin(\phi_{s+t}), \tag{S17}$$

where ϕ_s (ϕ_{s+t}) is the phase accumulated by the NV during the first (second) DD sequence (interrogation time) (Eq. S4), and the time s represents some arbitrary initial time. We model the number of photons detected coming from the NV as a Poisson distribution with a rate that depends on the NV state

$$Y_{s,t} \sim \mathrm{Pois}(\eta_{x_{s,t}}), \tag{S18}$$

$$X_{s,t} \sim \mathrm{Bernoulli}(p_{s,t}), \tag{S19}$$

where $\eta_{0,1}$ is the average photon count from the NV $m = 0,1$ state. Given the stochastic nature of the phases (ϕ_s) and the quantum nature of the system ($x_{s,t}$), the accessible distribution is the average photon count

$$P(y_t) = \mathop{\mathbb{E}}_{x_{s,t},\phi_s,\phi_{s+t}}[P(y_{s,t})]. \tag{S20}$$

4.1 Estimation

The FI (sec. 8) about the correlation function for correlation spectroscopy is given by

$$J_{\mathscr{C}(t),\mathscr{C}(t)} = \left(\frac{\partial \xi_t}{\partial \mathscr{C}(t)}\right)^2 \sum_{n=0}^{\infty} \frac{1}{2n!} \frac{(e^{-\eta_0}\eta_0^n - e^{-\eta_1}\eta_1^n)^2}{e^{-\eta_0}\eta_0^n(1-\xi_t) + e^{-\eta_1}\eta_1^n(1+\xi_t)}, \tag{S21}$$

$$\xi_t = e^{-\phi_{\text{rms}}^2}\sinh(\phi_{\text{rms}}^2\mathscr{C}(t)), \tag{S22}$$

where we denote $\phi_{\text{rms}} \approx 2\gamma B_{\text{rms}}\tau/\pi$ for small τ. The sum in Eq. S21 is bounded from above by $((1-\xi_t)(\xi_t + \coth((\eta_0 + \eta_1)/2)))^{-1}$, saturating in a scenario with full measurement contrast (i.e., $\eta_1 = 0$ and $\eta_0 > 0$). For small measurement contrasts this sum is approximately $c^2/(4\eta)$.

We use the sample mean to estimate the signal. The average photon count and variation are given by

$$\langle y_t \rangle = \eta - \frac{c}{2}\xi_t, \tag{S23}$$

$$\text{Var}[y_t] = \eta - \frac{c}{2}\xi_t + \frac{c^2}{4}(1-\xi_t^2), \tag{S24}$$

$$\tag{S25}$$

where $\eta = (\eta_0 + \eta_1)/2$ is the average photon count, $c = \eta_0 - \eta_1$ is the contrast. Thus, the information about $\mathscr{C}(t)$ (from the sample average of y) is given by

$$J_{\mathscr{C}(t),\mathscr{C}(t)} = \frac{1}{\text{Var}[y_t]}\frac{c^2}{4}\phi_{\text{rms}}^4 e^{-2\phi_{\text{rms}}^2}\cosh^2(\phi_{\text{rms}}^2\mathscr{C}(t)) \tag{S26}$$

$$= \frac{c^2}{4\eta + c^2}\phi_{\text{rms}}^4 + \mathscr{O}(\phi_{\text{rms}}^6), \tag{S27}$$

which coincides with the FI (Eq. S21) for small contrasts, and is relatively close (one order of magnitude) to the FI for large contrasts.

For the signal considered in this paper (Eq. S11), the information on the frequency is given by

$$j_{\delta,\delta} = J_{\mathscr{C}(t),\mathscr{C}(t)}t^2\sin^2(\delta t)C^2(t/T_\phi) \tag{S28}$$

$$= \frac{c^2}{4\eta + c^2}\phi_{\text{rms}}^4 t^2\sin^2(\delta t)C^2(t/T_\phi) + \mathscr{O}(\phi_{\text{rms}}^6) \tag{S29}$$

5 Synchronized measurements

We consider a measurement protocol as follows;

$$\begin{aligned}&\text{Initializing the NV to its ground state,}\\ &\text{pulses}: \ \mathrm{R}_y(\pi/2) - \mathrm{DD}(f_{\mathrm{DD}},\tau) - \mathrm{R}_{-x}(\pi/2),\\ &\text{state readout} + \text{clock readout.}\end{aligned} \tag{S30}$$

These measurements repeat in a synchronized fashion for each time $\tilde{\tau}$, and τ is the interrogation time. Accurately tracking the time between measurements enables us to correlate the measurement outcome in post-processing and estimate the signal.

The probability of the NV to be in the excited state is

$$q_s = \frac{1}{2} + \frac{1}{2}\sin(\phi_s) \tag{S31}$$

where ϕ_s is the phase accumulated by the NV during the DD sequence (interrogation time) (Eq. S4) performed at time s. We model the number of photons detected from the NV as a Poisson distribution with a rate that depends on the NV state

$$Y_s \sim \text{Pois}(\eta_{x_s}), \tag{S32}$$

$$X_s \sim \text{Bernoulli}(q_s), \tag{S33}$$

where $\eta_{0,1}$ is the average photon count from the NV $m = 0,1$ state.

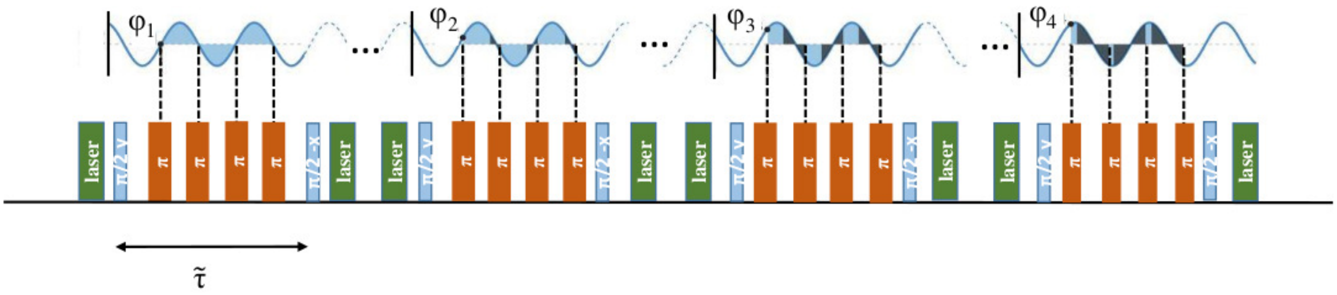

Figure S2. Synchronize/Qdyne measurement protocol. A sequence of initialization - CPMG dynamcial decoupling - readout, is repeated to gather information about the correlations in the sample.

5.1 Estimation

In order to estimate the correlation function we use the covariance between consecutive measurements,

$$\begin{aligned} \text{cov}(n_s, n_{s+t}) &= \frac{c^2}{4}\xi_t && \text{(S34)} \\ &= \frac{c^2}{4}\phi_{\text{rms}}^2 \mathscr{C}(t) + O(\phi_{\text{rms}}^4), && \text{(S35)} \end{aligned}$$

where ξ_t is as defined in Eq. S22. The sample covariance

$$S(t) = \frac{\tilde{\tau}}{T_{\text{tot}} - t} \sum_{s \in \{\tilde{\tau}, 2\tilde{\tau}, \ldots T_{\text{tot}} - \tilde{\tau}\}} n_s n_{s-t} - \eta^2 \quad \text{(S36)}$$

for different time differences (t) can no longer be considered an independent random variable, since it is calculated from a single time series $\{n_s\}$. The covariance between the sample covariance of different times is given by

$$\begin{aligned} \text{cov}(S(t_1), S(t_2)) =& \mathbb{E}[S(t_1)S(t_2)] - \mathbb{E}[S(t_1)]\,\mathbb{E}[S(t_2)] && \text{(S37)} \\ =& \delta_{t_1,t_2} \frac{\tilde{\tau}\eta^2}{T_{\text{tot}} - t}\left(1 + \left(\frac{c}{2\eta}\right)^2 (\xi_{t_1} - \eta\xi_0)\right) + && \text{(S38)} \\ &+ \frac{\tilde{\tau}2\eta}{T_{\text{tot}} - t}\left(\frac{c}{2}\right)^2 (\xi_{t_1+t_2} + \xi_{t_2-t_1}) + \\ &- \frac{\tilde{\tau}}{T_{\text{tot}} - t}\left(\frac{c}{2}\right)^4 \Big(\xi_{t_1+t_2} + \xi_{t_2-t_1} + \\ &+ \frac{1}{2}e^{-3\phi_{\text{rms}}^2}\cosh[2\phi_{\text{rms}}^2(\mathscr{C}[t_1] + \mathscr{C}[t_2])](e^{-\phi_{\text{rms}}^2\mathscr{C}[t_1+t_2]} + e^{-\phi_{\text{rms}}^2\mathscr{C}[t_2-t_1]}) + \\ &- \frac{1}{2}e^{-3\phi_{\text{rms}}^2}\cosh[2\phi_{\text{rms}}^2(\mathscr{C}[t_2] - \mathscr{C}[t_1])](e^{\phi_{\text{rms}}^2\mathscr{C}[t_1+t_2]} + e^{\phi_{\text{rms}}^2\mathscr{C}[t_2-t_1]})\Big) \\ =& \frac{\tilde{\tau}\eta^2}{T_{\text{tot}} - t}\left(\delta_{t_1,t_2}\left(1 + \frac{c^2\phi_{\text{rms}}^2}{4\eta^2}(\mathscr{C}[t_1] - \eta)\right) + \frac{c^2\phi_{\text{rms}}^2}{2\eta}(\mathscr{C}[t_1+t_2] + \mathscr{C}[t_2-t_1]) + \mathscr{O}(\phi_{\text{rms}}^4)\right) && \text{(S39)} \end{aligned}$$

where $t = \max(t_1, t_2)$, and the averaging is over the number of photons collected (n_s) over the distributions of $x_{s,t}, \phi_s, \phi_{s+t}$.

The information on $\mathscr{C}(t)$ (from the sample covariance) is given by

$$J_{\mathscr{C}(t),\mathscr{C}(t)} = \frac{1}{\text{cov}(S(t), S(t))}\frac{c^4}{16}\left(\frac{\partial\xi_t}{\partial\mathscr{C}(t)}\right)^2. \quad \text{(S40)}$$

For the signal that is considered in this paper (Eq. S11), the information on the frequency is given by

$$\begin{aligned} J_{\delta,\delta} =& \frac{c^4}{16}\sum_{s,w}\frac{\partial\xi_w}{\partial\delta}(([\text{cov}(S(t_1), S(t_2))]_{t_1,t_2})^{-1})_{s,w}\frac{\partial\xi_s}{\partial\delta} && \text{(S41)} \\ =& \frac{c^4}{16\eta^2}\sum_t \frac{T_{\text{tot}} - t}{\tilde{\tau}}\frac{\partial\xi_t}{\partial\delta}\frac{\partial\xi_t}{\partial\delta} + \mathscr{O}(\phi_{\text{rms}}^6). && \text{(S42)} \end{aligned}$$

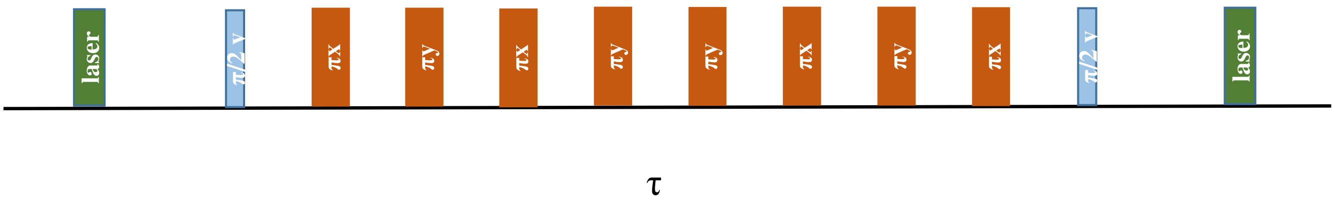

Figure S3. Power spectrum measurements protocol following an XY8 dynamical decoupling sequence. A 532 nm laser initializes and reads out the NV state. The sequence duration τ is varyied to scan the power spectrum of the sample.

6 Power spectrum measurements

In the scenario of power spectrum measurements we consider the measurement protocol;

$$\begin{aligned}&\text{Initializing the NV to its ground state,}\\ &\text{pulses}: \ \mathrm{R}_y(\pi/2)-\mathrm{DD}(f_{\mathrm{DD}},\tau)-\mathrm{R}_y(\pi/2),\\ &\text{state readout,}\end{aligned} \tag{S43}$$

such that the probability of the NV to be in the excited state is $p=\cos^2(\phi/2)$; thus, the average photon detection rate is given by

$$\langle y_\omega\rangle = \eta-\frac{c}{2}\exp(-\frac{1}{2}\phi_{\mathrm{rms}}^2\mathscr{S}_\tau(\omega)), \tag{S44}$$

$$\mathrm{Var}[y_\omega] = \langle y_\omega\rangle-\langle y_\omega-\eta\rangle^2+\frac{c^2}{4}, \tag{S45}$$

where $\phi_{\mathrm{rms}}^2=\gamma^2B_{\mathrm{rms}}^2T_\phi\tau$, and $\mathscr{S}_\tau(\omega)$ is the unit-less (normalized by $T_\phi\tau$) spectrum convoluted with a filter function of width τ^{-1}.

The measurement protocol in this scenario is similar to that of the correlation spectroscopy, with a different measurement basis and a longer interrogation time ($\tau\gtrsim T_\phi$), which could prove problematic if the coherence time of the sensor (T_2^{NV}) is short. Note that a strong field saturates the signal, which decays exponentially for large ϕ_{rms}^2. The power spectrum can be approximated as $\mathscr{S}_\tau(\omega)\propto 1-\alpha((\omega-\delta)T_\phi)^{n-1}-\alpha((\omega+\delta)T_\phi)^{n-1}$ for frequencies $\tau^{-1}\lesssim|\omega\pm\delta|\lesssim T_\phi^{-1}$ and $1<n<3$, where ω is the detuning of the DD frequency from the central frequency in the spectrum. For frequencies (ω) closer to the peaks ($\pm\delta$), the spectrum behaves as the shape of the filter function, which is usually quadratic. At a distance T_ϕ^{-1} from the peaks, the spectrum falls as ω^{-2}. For polynomial power $n>3$ the spectrum behaves quadratically. This means that the inverse interrogation time sets the resolution for this measurement, thus the interrogation time must be larger than δ^{-1}.

Under these restrictions ($\gamma^2B_{\mathrm{rms}}^2T_\phi\tau\lesssim 1<\delta\tau$ and $\tau\lesssim T_2^{\mathrm{NV}}$) , the information obtained about δ using the average number of photons is

$$j_{\delta,\delta}=\mathrm{Var}[y_\omega]^{-1}(0.5c\gamma^2B_{\mathrm{rms}}^2T_\phi\tau)^2e^{-\gamma^2B_{\mathrm{rms}}^2T_\phi\tau\mathscr{S}_\tau(\omega)}\left(\frac{\partial\mathscr{S}_\tau(\omega)}{\partial\delta}\right)^2. \tag{S46}$$

The (squared) derivative of the spectrum dictates how the information behaves. When the spectrum is smooth (i.e., the derivative with respect to ω is zero at the peak, $n>2$) the behavior of the (squared) derivative is similar to that of the Lorenzian case, but with a weaker dependence of $(\delta T_\phi)^{\min[2n-4,2]}$ at $\omega=0$. When the spectrum is sharp (i.e., the derivative is discontinuous at the peak, $1<n<2$) the (squared) derivative scales as $(\tau/T_\phi)^{4-2n}$, at $\omega=\delta-\tau^{-1}$.

For both the measurement resolution is set by τ which is limited by $\propto(\gamma^2B_{\mathrm{rms}}^2T_\phi)^{-1}$ or T_2^{NV}. An upper limit for the information is given when taking $(\gamma^2B_{\mathrm{rms}}^2T_\phi\tau)^2e^{-\gamma^2B_{\mathrm{rms}}^2T_\phi\tau\mathscr{S}_\tau(\omega)}\approx 1$,

$$j_{\delta,\delta}\lesssim\mathrm{Var}[y_\omega]^{-1}c^2T_\phi^2\begin{cases}(\delta T_\phi)^{\text{min[2n-4,2]}} & ,n>2\\ (\tau/T_\phi)^{4-2n} & ,1<n<2\end{cases} \tag{S47}$$

This means that for $n<2$ the information is independent of δ and the resolution is set by τ^{-1}, for $n>2$ there is a reduced "penalty" for small δT_ϕ.

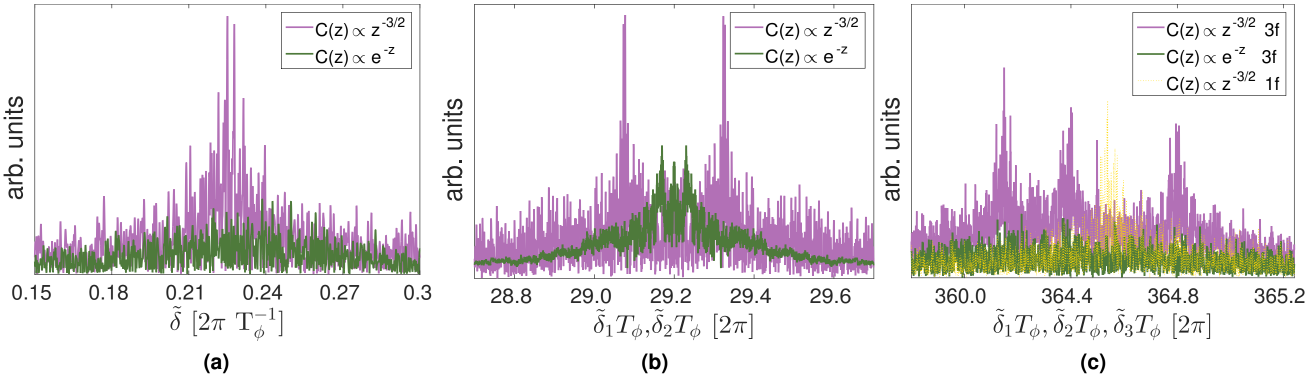

Figure S4. Average of 400 CFT of measurement vectors for the cases of one frequency (a), two frequencies (b) and three frequencies (c), with the same parameters as used in the main text to generate the corresponding histograms. In purple, the case of $C(z^{-1.5})$ Eq. S13 correlations whereas in green the correlations are exponential, which do not allow for frequency resolution.

7 Fourier Transform examples

Correlation function fitting has the disadvantage that the noise produces displacements on the parameters, which manifest as a widening of histograms, but are less prone to local minima. In Fourier transform analysis, however, noise reflects appears as extra peaks that require more computation time to be avoided. Nonetheless, a direct Fourier transform of the signal can produce more visual results. In Fig. S4 we present results for Continuous Fourier Transform (CFT) for the cases of signals containing one (a) two (b) and three (c) frequencies. Each CFT is calculated as

$$CFT(\omega_i) = \sum_j s_j e^{-i\omega_i t}. \tag{S48}$$

Each plot is the average of 400 measurement vectors, where extended correlations allow for frequency resolution while exponential correlations produce a spectrum without defined peaks.

8 Fisher Information

The Fisher Information (FI) matrix (for parameters θ_i, θ_j) is defined as

$$J_{i,j} = \underset{L(\theta)}{\mathbb{E}} \left[\frac{\partial \log(L(\theta))}{\partial \theta_i} \frac{\partial \log(L(\theta))}{\partial \theta_j} \right], \tag{S49}$$

where L is the probability function, and θ is a set of parameters that defines the probability.

References

1. Cywiński, L., Lutchyn, R. M., Nave, C. P. & Das Sarma, S. How to enhance dephasing time in superconducting qubits. Phys. Rev. B **77**, 174509, DOI: 10.1103/PhysRevB.77.174509 (2008).
2. Rotem, A. et al. Limits on spectral resolution measurements by quantum probes. Phys. Rev. Lett. **122**, 060503, DOI: 10.1103/PhysRevLett.122.060503 (2019).
3. Cohen, D. et al. Utilising nv based quantum sensing for velocimetry at the nanoscale. Sci. Reports **10**, 5298, DOI: 10.1038/s41598-020-61095-y (2020).
